# Thermo-optical tweezers based on photothermal waveguides

**DOI:** 10.1038/s41378-024-00757-7

**Published:** 2024-09-02

**Authors:** Fuwang Li, Jian Wei, Xiaomei Qin, Xue Chen, Dawei Chen, Wentao Zhang, Jiaguang Han, Libo Yuan, Hongchang Deng

**Affiliations:** 1 Photonics Research Center, School of Optoelectronic Engineering, Guilin University of Electronic Technology, Guilin, 541004 China; 2 School of Mechanical and Electrical Engineering, Guilin University of Electronic Technology, Guilin, 541004 China; 3 Guangxi Key Laboratory of Optoelectronic Information Processing, School of Optoelectronic Engineering, Guilin University of Electronic Technology, Guilin, 541004 China; 4 Center for Terahertz Waves and College of Precision Instrument and Optoelectronics Engineering, and the Key Laboratory of Optoelectronics Information and Technology (Ministry of Education), Tianjin University, Tianjin, 300072 China

**Keywords:** Microfluidics, Micro-optics

## Abstract

Field-controlled micromanipulation represents a pivotal technique for handling microparticles, yet conventional methods often risk physical damage to targets. Here, we discovered a completely new mechanism for true noncontact manipulation through photothermal effects, called thermal-optical tweezers. We employ a laser self-assembly photothermal waveguide (PTW) for dynamic microparticle manipulation. This waveguide demonstrates superior photothermal conversion and precision control, generating a nonisothermal temperature field. The interaction of thermal convection and thermophoresis within this field creates a microfluidic potential well, enabling noncontact and nondestructive particle manipulation. By varying the path of PTWs in lithography and manipulating laser loading modes, diverse manipulation strategies, such as Z-shaped migration, periodic oscillation, and directional transport, are achievable. Our innovative noninvasive micromanipulation technology minimizes not only physical damage to target objects but also enables precise and diverse manipulation of micro entities, opening up new avenues for the photothermal control of cells and biomolecules.

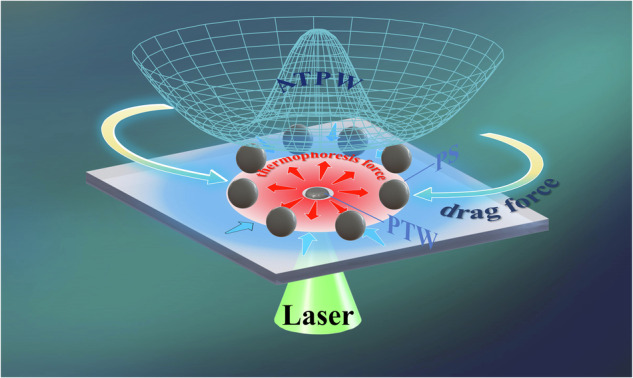

## Introduction

Field-controlled micromanipulation techniques, which are extensively applied in various fields, such as cell migration, drug delivery, microassembly, and micro nano technology, propose methods for precisely manipulating microparticles using external force fields. Notably, among these applications, migrating microparticles in a controlled and noncontact manner is in high demand. Since Ashikn first observed the manipulation of microparticles by external optical forces^1^, the noncontact application of optical tweezers has significantly advanced. Since then, similar technologies have emerged for manipulating colloidal particles and biological objects^2–6^, including magnetic tweezers^7–9^, opto-refrigerative tweezers^10,11^, and acoustic fluid and microfluidic techniques^12–15^. However, these technologies are often limited by poor specificity^16,17^, low spatial resolution^18^, and potential photothermal damage^19^, posing challenges to precise, damage-free manipulation of microparticles. To overcome these limitations, an effective strategy, termed thermo-optical tweezers (TOTs)^20^, was introduced. Specifically, photothermal materials (including gold, silver, indium tin oxide, and nanoarray gold films) absorb heat via the photothermal effect and subsequently generate a nonisothermal temperature field in the fluid^21–23^, thereby enabling the manipulation of microparticles through thermal convection and thermophoresis^24,25^. It should be noted that, compared to optical tweezers, TOT offers a larger controllable range, fewer requirements for particle properties, and reduced photothermal degradation, thus providing significant advantages in terms of low damage and noncontact manipulation. In recent years, researchers have explored the use of temperature fields instead of a strongly focused laser for particle manipulation^26–28^. However, these methods face limitations in manipulating microparticles via diverse routes.

Here, we introduce novel TOTs utilizing laser-induced photothermal waveguide (PTW) self-assembly for noncontact micromanipulation of particles. The waveguide demonstrates remarkable photothermal conversion and control, inducing dominant forces such as drag force in the far-field and thermophoresis force in the near-field on particles, thereby generating a stable microfluidic potential well for controllable micromanipulation of particles. This combination of near-field and far-field dominant forces enables the manipulation of not only particles near the waveguide but also the attraction and relocation of distant particles to preset positions, as well as the manipulation of particles with various materials, including negative refractive index particles and irregular substances. This novel technology represents a new era in the application of TOTs in field-controlled micromanipulation.

## Results

**Fig. 1:**
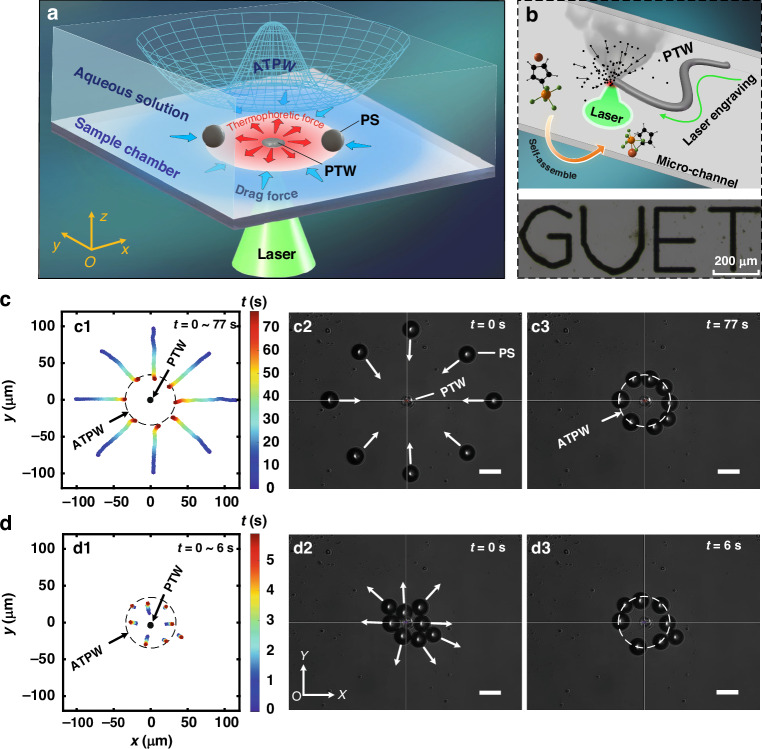
Dynamic manipulations of the PS particles driven by the TOT. **a** The schematic diagram illustrates PS particle manipulation through the combined action of the thermophoretic force and drag force in the surroundings of the PTW. Initially, under the photothermal effect of the laser-induced PTW, a drag force toward the PTW is generated by the temperature gradient, as indicated by the blue arrow. When the PS particles approach the PTW, they are subjected to an outward thermophoretic force, as shown by the red arrow. ATPW is formed by these two forces, leading to the trapping and manipulation of particles. By modifying the structure of the PTW and the laser loading modes, the proposed potential well enables versatile and dynamic manipulation of particles. **b** The schematic diagram presents the laser-induced PTW self-assembly and the experimental PTW pattern labeled ‘GUET’. The yellow arrows indicate the two states before and after the self-assembly of PTW, while the green arrows represent the photolithography trajectory. **c** The trajectory (colored curve) of the PS particles initially positioned away from the PTW, starting at (c2) and ending at (c3). Particles are attracted toward the center and eventually stabilize at the ATPW (*r* = 34 μm, dotted circle). **d** The outward flow of PS particles, which were initially placed close to the PTW. All the particles move outward to the ATPW region. The black dot in the center represents the PTW, and the white arrows indicate the movement direction of the PS particles. The incident beam power in the experiment, measured at the objective lens’s back aperture, is *P* = 7.3 mW. Scale bars: 50 μm

The dynamic manipulation of microparticles driven by TOT induced by the laser source was experimentally investigated. As shown in Fig. 1a, the experimental module features a rectangular sample chamber (10 × 10 × 1 mm^3^) filled with an aqueous solution containing 0.1% polystyrene particles (PS, 20 μm, Wuxi Rigor Technology Co., Ltd.). Figure 1b illustrates the self-assembly of the photoinitiator GR-261 (PGR, CAS:32760-80-8, Hubei Gurun Technology Co., Ltd.) into a crystalline state, which is crucial for PTW fabrication using the laser-induced PTW self-assembly method. The proposed lithography technology enables the production of precisely controlled PTW patterns. The PTW absorbs the appropriate incident light (see Supplementary Fig. S2) from the focused laser source and then produces a localized hot spot at the bottom of the sample chamber without any bubbles. Through the photothermal conversion of PTW, a nonisothermal temperature field is induced in aqueous solution, resulting in a synergistic effect between the drag force and thermophoresis force acting on the PS particles, thus creating an annular trapping potential well (ATPW). Interestingly, we observed that the PS particles exhibited two typical dynamic migrations, namely, inward and outward transport, enabling stable trapping of the particles at the ATPW’s lowest point, as illustrated in Fig. 1c, d (see Supplementary Movie S1). For PS particles initially placed far from the PTW, the drag force attracts them toward the center along the radial direction, ultimately stabilizing them at the ATPW, as shown in Fig. 1c. Conversely, if PS particles are initially close to the PTW, they are repelled by the thermophoretic force and subsequently trapped in the same region of the ATPW, as shown in Fig. 1d. This behavior differs from that described in the literature^21–23^, where most particles could only move in one direction and gather toward the center. However, our results demonstrate that TOT can manipulate PS particles in both directions, depending on their initial positions. More importantly, all the particles are ultimately trapped in the same ATPW region, with a radius *r* = 34 μm, in a noncontact and nondestructive manner.

**Fig. 2:**
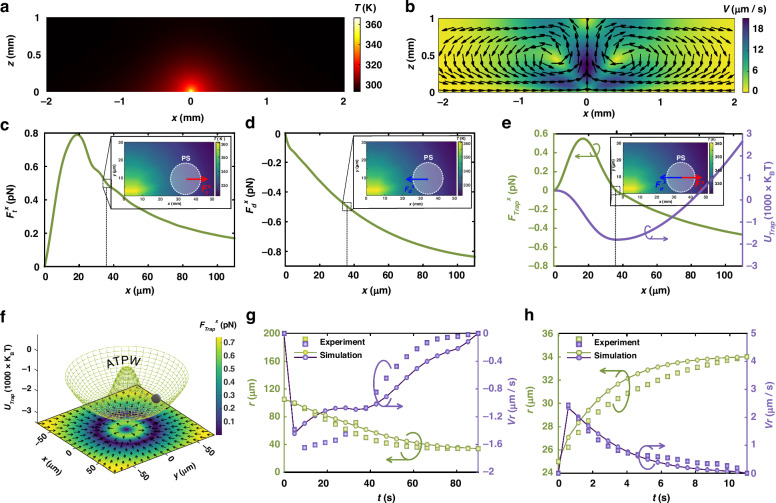
The particle migration mechanism of TOTs. **a** Local temperature distribution of the TOT in the *x*‒*z* plane. **b** Local velocity distribution of the microfluid in the *x*‒*z* plane. **c** Thermophoretic force distribution acting on the PS particle along the *x* axis. Inset: The thermophoretic force ${F}_{t}^{x}$ acting on the PS particle. **d** Drag force distribution acting on the PS particle along the *x* axis. Inset: The drag force ${F}_{{\rm{d}}}^{x}$ acting on the PS particle. **e** Distribution of the trapping force and trapping potential along the *x* axis. Inset: The trapping force exerted on the PS particle at the potential well. **f** Distribution of the trapping force (color) and ATPW (mesh) acting on the PS particle on the *x‒y* plane, where it is observed that the PS particle is trapped at the lowest point of the ATPW. **g**, **h** Average displacements and radial velocities of the PS particle with respect to time. The experimental results are denoted by purple and green squares, respectively, while the simulated results are illustrated by purple and green circle lines. The color bar indicates the amplitude, and the arrows denote the direction

To further investigate the particle manipulation mechanism of TOTs, we propose a physical model that considers the thermal flow and thermophoresis acting on the particles (see Supplementary for the physical model). Figure 2a, b depict the resulting distributions of local temperature and velocity fields driven by the TOT. The local heat source is shown to generate a transient nonisothermal temperature field and a significant local temperature gradient, subsequently inducing a thermal convection flow that circulates the surrounding fluid. During this process, microparticles in the nonisothermal fluid experience two primary forces in the horizontal direction, namely, drag and thermophoretic forces, and the gravitational force is negligible. The drag force, governed by Stokes’ drag law due to thermal flow, propels the particles in the direction of fluid velocity. Conversely, the thermophoretic force, induced by the local temperature gradient, drives the particles from hotter to colder regions. Figure 2c, d shows the magnitudes of these two forces on the PS particle at various positions along the *x* axis. Notably, these two forces act in opposite directions, indicating that the thermophoretic force near the waveguide can counteract the drag force. Consequently, the interplay of these directional forces results in the trapping of microparticles due to force equilibrium. Figure 2e shows the distributions of the trapping force and trapping potential along the *x*-axis. The results reveal that PS particles with a diameter of 20 μm can achieve zero trapping force at *r* = 34 μm, with the corresponding minimum trapping potential of at least −2000 k_B_T. This explains why the particles are trapped within the trapping potential well. From a top view, Fig. 2f illustrates the distributions of the trapping force and trapping well on the *x*‒*y* plane. Here, a black arrow signifies the direction of the trapping force, pointing toward the lowest region, corresponding to the lowest point of the potential well. The PS particles are stably trapped^29,30^ at the ATPW, and the trap stiffnesses of the outward (positive) and inward (negative) trapping forces are ~35 fN/µm and 6 fN/µm (see Supplementary Fig. S3A), respectively. Consequently, the response of PS outward movement is faster than that of inward attraction, which is consistent with the experimental results. Furthermore, we observed that in the *z*-directional force field, there is a trapping force on the order of piconewtons (see Supplementary Fig. S3B) near the thermo-optical tweezers (PTWs). We systematically investigated the experimental results of particles initially located both outside and inside the ATPW. Figure 2g, h show how the average displacement and radial velocity of the microparticles vary with time. The results suggest that if the initial position of the particle is beyond the ATPW, the heat source has an attractive effect on it. Conversely, the heat source repels nearby particles toward the ATPW region. Moreover, the simulated results agree well with the experimental results shown in Fig. 1c, d, which indicates that our proposed model accurately explains the particle trapping process using TOTs.

**Fig. 3:**
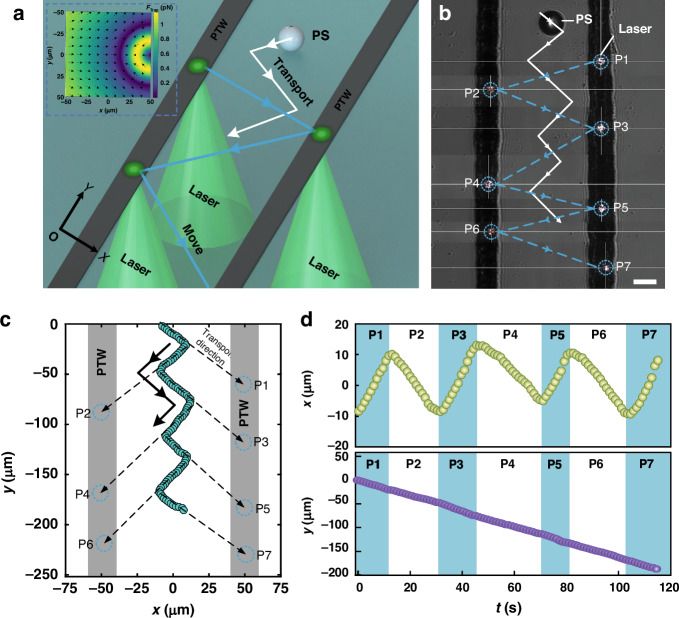
Z-shaped migration properties of PS particles driven by TOT. **a** Schematic diagram of PS particle migration through double-track PTWs with a spacing of 100 μm. The solid white line depicts the trajectory of the PS particle. The solid blue line depicts the moving track of the SFL. Inset: Trapping force distributions of the SFL. The color bar indicates the amplitude, and the arrows denote the directions of the trapping force. **b** Experimental migration results of a single particle. The solid white line represents the trajectory of the PS particle. The dashed blue line indicates the moving track of the SFL. The laser is focused on the center of the dashed blue circle. The incident beam power is 9.8 mW. Scale bar: 25 μm. **c** Transport of the PS particle on the *x‒y* plane. The circles with dashed blue lines represent the laser positions. The solid green circles mark the positions of the PS particles. The black arrows show the direction of PS particle transport. **d** The trajectories vary with time along the *x*- and *y*-axes

Here, we introduce three oriented routes for a single particle manipulated by the TOT system, each employing different heat source loading modes. First, for a single microparticle following a Z-shaped path, double-track PTWs are applied, and a single-focus laser (SFL) alternates between the two PTW tracks from the P1 to P7 positions, as illustrated in Fig. 5a, b (refer to Supplementary Movie S3). According to the previously described model, the particle is attracted to the ATPW. Thus, when the laser transitions from one PTW track to another, the particle changes its direction of movement accordingly. The trajectories, varying over time on the *x* axis and *y*-axis, are depicted in Fig. 3c, d, demonstrating how the single particle follows the line between itself and the laser to form a Z-shaped path. Notably, the particle’s movement in the *y*-direction is nearly linear, moving at a speed of 1.61 μm/s, while its trajectory along the *x* axis is sinusoidal. This insight offers novel possibilities for manipulating cells and biological macromolecules in various fields, including life sciences, biomedicine, microassembly, and chemical research.

**Fig. 4:**
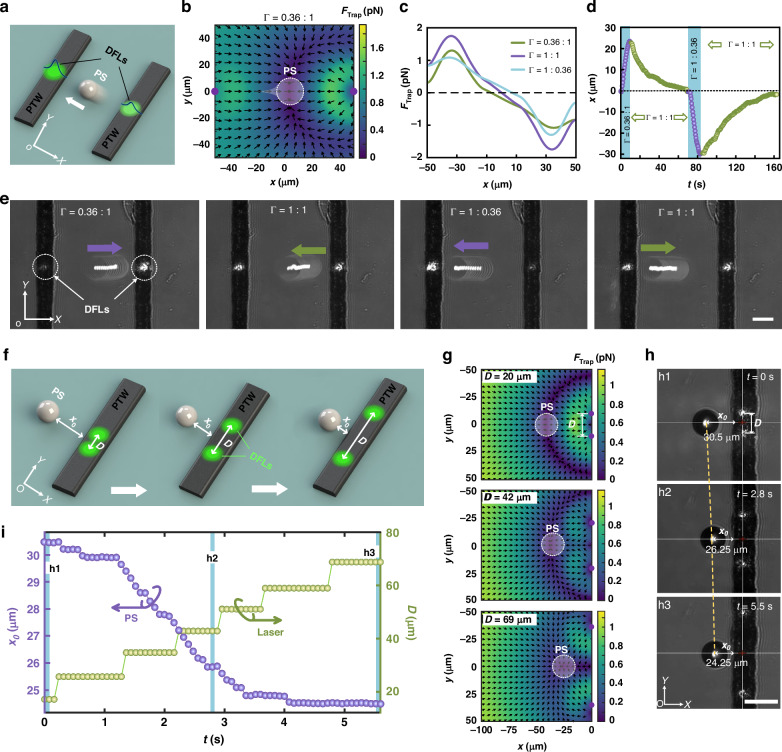
Study on the horizontal migration characteristics of TOTs by DFLs. **a** Schematic diagram of the horizontal migration of PS particles controlled by DFLs with varying optical powers. **b** Trapping force distributions with an optical power ratio of 0.36:1. The color bar indicates the amplitude, and the arrows denote the directions of the trapping force. **c** Axial trapping force curves on the *x* axis for different optical power ratios. **d** Particle displacement in the *x* axis over time. **e** Migration of the PS particle in the horizontal direction is achieved by alternating the optical powers. The combined power of the two lasers is 14.6 mW. **f** Schematic illustration of the horizontal migration of a single particle through adjustable-spacing DFLs on a single-track PTW**. g** Trapping force distributions on the *x*‒*y* plane for DFL spacings of D = 20, 42, and 69 μm. The purple dots symbolize the lasers. The white dotted circles indicate PS particles trapped in the force potential wells. The color bar indicates the amplitude, and the arrows denote the directions of the trapping force. **h** Experimental results of a PS particle trapped by TOT with varying DFL spacings. *x*_*0*_ denotes the horizontal distance of the particle from the PTW. **i** Displacement *x*_*0*_ of the particle and spacing D of the DFLs varying with time. Each focused laser has an incident power of 11.1 mW. Scale bar: 25 μm

Second, by modulating the optical power ratios of double-focus lasers (DFLs), the particles can be induced to oscillate back and forth in a manner similar to horizontal oscillation. This method promotes the horizontal migration of a single particle through TOTs, as demonstrated in Fig. 4a. The underlying mechanism involves changing the power ratio (*Γ* = P_1_/P_2_), as shown in Fig. 4b, which leads to an off-center potential well-induced by the laser-induced temperature gradient and drives a single particle toward the region of the minimum trapping potential. Figure 4c displays the axial capture force distribution at *y* = 0, with varying power ratios of 0.36:1, 1:1, and 1:0.36. The results indicate that when the optical powers of the two lasers are equal, the particle is trapped at the center; if they differ, the particle gravitates toward the side with higher power. Furthermore, by alternating the optical powers of the two lasers, the particle can oscillate back and forth along the *x*-axis, as demonstrated in Fig. 4d, e. (refer to Supplementary Movie S4). When the laser power ratio changed from 1:1 to 0.36:1, the particle initially at the center (*x* = 0 μm) migrated toward the higher power position by 23.2 μm within 10 s. Upon the power ratio returning to 1:1, the particle-stabilized back at its initial position (*x* = 0 μm) in 59 s. Similarly, when the laser power ratio was reversed, a comparable reciprocating motion occurred. This observation shows that transport toward the center of the potential well takes longer, which aligns with the fact that the particle experiences less force near the potential well, confirming our simulation predictions.

Figure 4f shows another way to manipulate a PS particle horizontally by using TOTs with a single-track PTW. As depicted in Fig. 4g, the existing model suggests that when the spacing *D* between two lasers on a single-track PTW increases, the particle initially at the symmetry center of the DFLs will be driven by the altered trapping potential well and move along the *x*-axis toward the PTW side. Similarly, when the DFL spacing decreases, the particle will move outward, away from the PTW horizontally. Figure 4h illustrates the movement of a PS particle in the experiment as the spacing *D* increases (refer to Supplementary Movie S5). When the spacing of the DFLs increases from 20 μm to 69 μm in a step function, the particle, which initially stabilizes at *x*_*0*_ = 30.5 μm, moves to *x*_*0*_ = 24.5 μm. As depicted in Fig. 4i, the particle moves linearly along the *x*-axis at a speed of 0.77 μm/s from 1.5 s to 3.5 s. In the final stage, it becomes trapped and remains stationary near *x*_*0*_ = 24.5 μm.

**Fig. 5:**
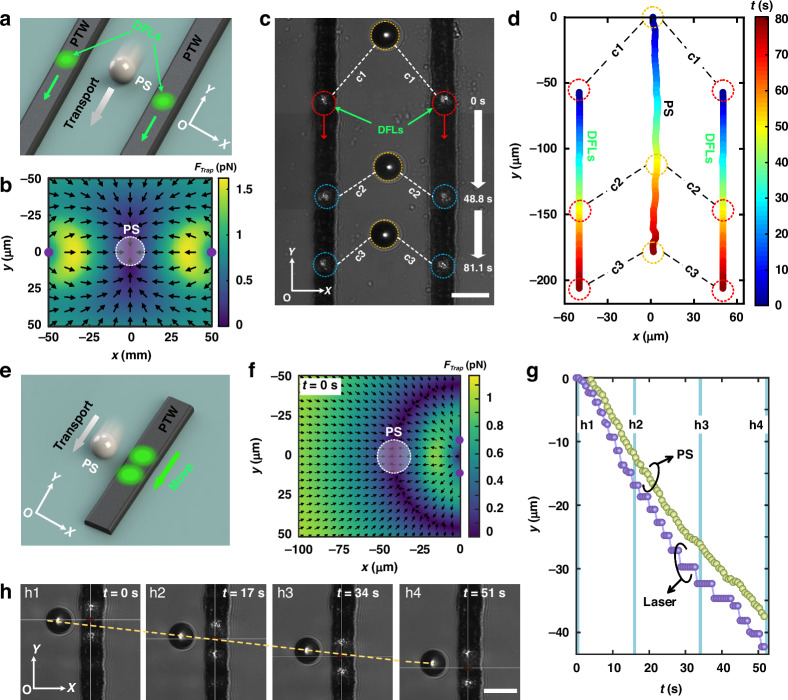
Particle vertical migration properties of TOTs by DFLs. **a** Schematic illustration of the vertical migration of a single particle through two parallel moving DFLs. **b** The trapping force distributions of TOT with DFLs on double-track PTWs. The color bar indicates the amplitude, and the arrows show the directions of the trapping force. The optical power of each laser is 12.0 mW. **c** Experimental results of the vertical migration of TOT particles. The red arrow indicates the movement direction of the lasers. Red and blue dotted circles mark the positions of the lasers. The yellow dotted circles represent the positions of the PS particles at 0 s, 48.8 s, and 81.1 s. **d** The moving trajectories of the PS and DFLs over time on the *x*‒*y* plane. **e** Schematic diagram of vertical particle migration by DFLs along a single PTW. **f** The trapping force distribution of TOTs with DFLs on a single-heeled PTW. The white dotted circles indicate PS particles trapped in the force potential wells. **g** PS particle and laser change curves with time along the *y*-direction. **h** Experimental results of particles moving in the *y*-direction. The optical power of each laser is 11.1 mW. Scale bar: 25 μm

Third, for single particle vertically directed transport of TOT, as illustrated in Fig. 5a, we utilize two parallel moving DFLs with identical optical powers. Initially, the particle starts in an upper position and then quickly moves to the trapping potential well near the center, as depicted in Fig. 5b. Since the trapping force is weaker near the symmetry center between the two heat sources, particles in the upper position may halt off-center at the upper position labeled c1. Subsequently, it follows the descending DFLs along two line-patterned PTWs from positions c1 to c3, as shown in Fig. 5c, d (refer to Supplementary Movie S6). This method can controllably trap particles at a specific position by using TOTs and then transport them vertically to a predetermined location, which is highly important for accurately manipulating micro/nanoscale objects. Similarly, with the downward movement of the DFLs, it is also easy to move particles along the *y* axis. As shown in Fig. 5e, when the DFLs are located on a single-track PTW, a stable trapping potential well can be formed (see Fig. 5f), making the manipulation of particles more stable. Consequently, downward-moving DFLs with uniform velocity on a single-track PTW are employed to determine the vertical trajectory of the particle. As depicted in Fig. 5g, h, as the DFLs decrease in the negative *y*-direction, a PS particle moves linearly along the vertical axis at a speed of 0.7 μm/s (see Supplementary Movie S7). This synchronized motion of the particles with the DFLs is not only closely aligned with their path but also shows the robust and stable ability to manipulate particles.

More importantly, we discovered that the TOT is not restricted by refractive index variations and can dynamically manipulate fluorinated liquid particles with negative refractive indices (refer to Supplementary Fig. S6 and Movie S8). However, traditional optical tweezers are limited to capturing particles with positive refractive indices, demonstrating that TOT is a more versatile micro/nanoparticle manipulation device. Additionally, the adaptability of the TOT system to different particle shapes was investigated (refer to Supplementary Fig. S7 and Movie S9). The results demonstrate that TOT can also capture and manipulate irregular particles, thereby expanding its applications in biomedicine.

## Discussion

In summary, we proposed a TOT system based on a novel laser-induced PTW self-assembly method with various patterned PTWs on the bottom for the dynamic manipulation of microparticles. As the PTW absorbs the laser source to create a hot spot, it induces a nonisothermal temperature field and generates drag and thermophoresis forces acting on the particle. Interestingly, we found that under a fixed laser, PS particles initially located at different positions exhibit two typical dynamic migrations: inward and outward. However, regardless of their initial positions, the particles eventually become trapped in the same ATPW region. This observation obviously differs from that of an opto-refrigerative tweezer system. We further proposed a physical model to quantitatively investigate the particle migration mechanisms. The balancing forces facilitate the trapping of the particle within the trapping potential well. Subsequently, DFLs are employed to precisely control a single particle. The results reveal that particles from different initial positions all converge toward the center of the trapping potential well, aligning with the manipulation mechanisms of the TOT system. Compared to previous studies, our findings offer novel photothermal insights into the precise noncontact manipulation of particles, thereby preventing photothermal damage.

To manipulate a single particle along an oriented route, single- and DFLs with varying positions and optical powers are utilized. Three manipulation routes were experimentally investigated: Z-shaped migration, particle oscillation, and directed transport. For particles moving in a Z-shaped path, an alternating SFL on a double-track PTW is applied. If the optical power ratios or the distances of the DFLs are changed, the particle can oscillate horizontally. Otherwise, if the DFLs are moving down in a single or two parallel tracks, the particle can be transported vertically. This indicates that the particle trajectories can be precisely controlled by designing the structures of the PTWs and the loading modes of the heat sources. Additionally, the TOT system can precisely capture particles with negative refractive indices and irregular shapes near the PTW in a non-contact manner. Thus, our work offers entirely new photothermal insights into precise noncontact particle manipulation.

Our findings enhance the fundamental understanding of suspended particles in a nonisothermal temperature field and broaden the scope for biological applications involving field-controlled micromanipulation. For instance, this approach plays a crucial role in single-cell Raman detection, obtaining rich intrinsic information about target cells. Additionally, this method can be applied to the collection of cells, sorting of bacteria and cells, delivery of drug reagents, monitoring of the apoptosis process of tumor cells, and prediction of drug resistance in lung cancer cells, thereby broadening its potential in the biomedical and life science fields.

## Materials and methods

### Chemicals

The photoinitiator GR-261 (CAS: 32760-80-8, supplied by Hubei Gurun Technology Co., Ltd.), monodisperse PS microspheres (PS, 20 μm, from Wuxi Rigor Technology Co., Ltd.), pure water (provided by Jiangsu Xizhimeng Trading Co., Ltd.), fluorinated liquid (Novec 7500, manufactured by Minnesota Mining and Manufacturing Co., Ltd.), and PVA surfactant (CAS: 9002-89-5, produced by Shanghai Aladdin Biochemical Technology Co., Ltd.) were utilized in this study.

### Synthesis of PTW

A PTW was synthesized using a laser, which is crucial for PTW fabrication via the laser-induced PTW self-assembly method. Initially, 1.5 g of PGR powder was added to 20 mL of deionized (DI) water and stirred for 5 min to create a PGR solution. Next, 0.1 mL of this PGR solution was transferred into a rectangular sample chamber (10 × 10 × 1 mm³). A 65 mW laser was then focused on the bottom of the channel. Since PGR is a photoinitiator, it can absorb radiant energy in the ultraviolet and visible light spectrum. Upon irradiation with a 532 nm laser, cationic polymerization is triggered, causing the PGR to self-assemble into a crystalline state. This process forms a solid PTW at the laser’s focal point, akin to a laser, which is crucial for PTW fabrication using laser-induced PTW self-assembly. As the laser moves along the underside of the channel, it creates arbitrary PTW patterned structures (see Supplementary Fig. S8). Subsequently, the remaining PGR solution was rinsed with DI water five times.

### Sample preparation

The sample chamber was constructed from two glass slides, one of which had a hollow measuring 10 × 10 mm in the middle. After laser irradiation, which is crucial for PTW fabrication using the laser-induced PTW self-assembly method, various PTWs patterned on the bottom were applied, 20 μL of a PS solution was mixed with 20 mL of DI water, and sonicated for 15 min to form a 0.1% aqueous PS particle solution. This solution was then introduced into the sample chamber. Upon absorbing energy from the laser, the PTW generated a localized hot spot. This created a nonisothermal temperature field that facilitated the trapping of the PS particles. For the preparation of fluorinated oil microparticles, 5 mL of fluorinated liquid and 5 mL of polyvinyl alcohol were combined in 5 mL of DI water and homogenized for 1 min.

### Experimental system

The TOT system comprises a laser source module, an observation module, and a sample chamber experimental module. The laser source module, which utilizes holographic beams, is illustrated by the green-light paths in Supplementary Fig. S1. A 532 nm wavelength laser beam (MGL-N-532, 3 W, Changchun New Industries Optoelectronics Technology Co., Ltd.) is reflected by a dielectric mirror (M1) and subsequently collimated and expanded by lenses (L1 and L2). This process ensures that the spatial light modulator (SLM) reflective plane (GCI-770402, 60 Hz, Daheng New Epoch Technology, Inc.) is adequately filled. A half-wave plate aligns the laser beam’s polarization with the polarization axis of the SLM. The modified light is then reflected by another dielectric mirror (M2) and directed onto the SLM at a slight incident angle. A 4 f lens system consisting of two lenses (L3 and L4) images the holograms produced by the SLM onto the dichroic mirror (DCM). This image is then relayed from the DCM to the back focal plane of the microscope objective, forming a micron-scale laser source. Additionally, SLM can be employed to modulate multiple focused laser sources.

### Observation system

The observation module consists of a red LED light source and lens L5 to illuminate the experimental module, as depicted by the red light paths in Fig. S1. Dielectric mirrors (M3, M4, M5, and M6) are strategically placed in the optical path to modify the direction of the light. The DCM is characterized by high reflectivity for green light and high transmittance for red light, enabling the dynamic behaviors of microparticles to be captured by a CCD camera (MER2-135-208U3M, 208fps, Beijing Daheng Image Vision Co., Ltd.) through a MO and lens L6. The corresponding displacements, velocities, and trajectories of the microparticles under varying incident optical powers are then analyzed using image processing methods.

### Supplementary information

supplementary

Dynamic manipulation of eight PS particles driven by TOT

Dynamic manipulation of a single PS particle in different positions by DFLs

A single particle migrates in a Z-shaped path by an alternative SFL on the double-track PTW

A single particle migrates in the horizontal direction by the DFLs with differing optical power ratios

A single particle migrates in the horizontal direction by the moving DFLs with adjustable distance on a single-track PTW

A single particle migrates in the vertical direction by two parallel moving DFLs

A single particle migrates in the vertical direction by the downward-moving DFLs with the same velocity along the single-track PTW

Real-time trapping of fluorinated oil microparticles (diameter: 30 μm) with negative refractive index by the TOT

Real-time trapping of bamboo fiber particle (BFP) with irregularly shaped index by the TOT
