# Supplementary material for: Thermo-optical tweezers based on photothermal waveguides
Source: Microsyst Nanoeng. 2024 Sep 2;10:123. doi: 10.1038/s41378-024-00757-7 (PMC11368956; doi:10.1038/s41378-024-00757-7)
Supplement: Supplementary file 1 — supplementary [file 41378_2024_757_MOESM1_ESM.docx]

**Supplementary Information:** **Thermo-optical tweezers based on photothermal waveguides**

**Fuwang Li^1^, Jian Wei^1^, Xiaomei Qin^1^, Xue Chen^2^*, Dawei Chen^1^, Wentao Zhang^3^, Jiaguang Han^3,4^, and Libo Yuan^1^, Hongchang Deng^1^***

1. Photonics Research Center, School of Optoelectronic Engineering, Guilin University of Electronic Technology, Guilin 541004, China.

2. School of Mechanical and Electrical Engineering, Guilin University of Electronic Technology, Guilin 541004, China.

3. Guangxi Key Laboratory of Optoelectronic Information Processing, School of Optoelectronic Engineering, Guilin University of Electronic Technology, Guilin 541004, China.

4. Center for Terahertz Waves and College of Precision Instrument and Optoelectronics Engineering, and the Key Laboratory of Optoelectronics Information and Technology (Ministry of Education), Tianjin University, Tianjin, 300072, China.

*Corresponding author. Email: hcdeng@guet.edu.cn

**Supplementary Figures**

**
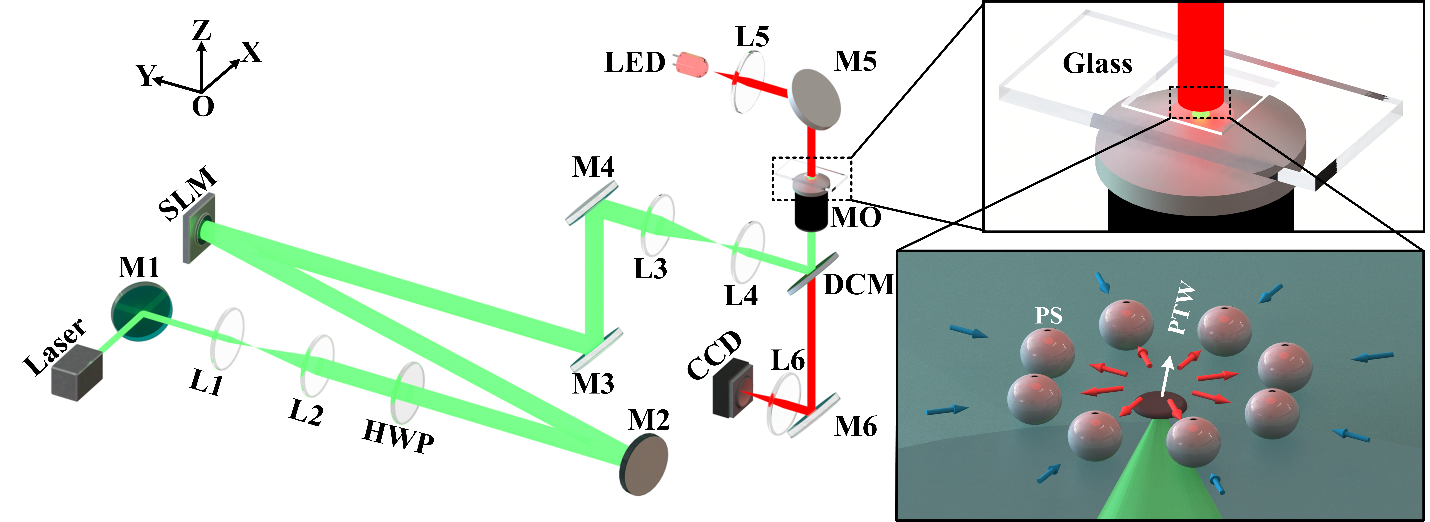
**

**Fig. S1. Experimental Setup of the TOT System.** Components include M for dielectric mirrors, L for lenses, HWP for the half-wave plate, SLM for the spatial light modulator, DCM for the dichroic mirror, and MO for the microscope objective. Insets: This schematic diagram of the TOT system illustrates the manipulation of PS particles. The red and blue arrows indicate the thermophoretic and drag forces acting on the PS particles, driven by the thermal convection flow.

**Physical Model**

In this paper, we propose a physical model that takes into account the thermal flow driving particle motion and the thermophoresis acting on the particles. When a laser ($\lambda=532$nm) is incident on the PTW, the optical energy is absorbed and converted into a distribution of luminous intensity, denoted as *I*,

$I=\frac{2P}{\pi w^{2}(z)}\exp(-2\frac{r^{2}}{w^{2}(z)})$, (1)

where $r$ and $z$ are the cylindrical coordinates, *P* is the optical power of the laser. Here, $w(z)$ is the beam radius, which can be defined as:

$w(z)=w_{0}\sqrt{1+(\frac{\lambda z}{\pi w_{0}^{2}})^{2}}$, (2)

where $w_{0}$ represents waist radius, $\lambda$is the wavelength.

After absorbing incident optical power from the laser source, the PTW could generate a heat flux $H_{t}$, which is proportional to the distribution of luminous intensity,

$H_{t}=\varepsilon I$, (3)

where $\varepsilon$is the emissivity coefficient.

Thus, heat flux generates a transient non-isothermal temperature field in the aqueous solution, inducing the thermophoresis force and drag force. Thermophoretic force is acting on the particle due to the temperature gradient $\nabla T$. In a continuum flow, the thermophoretic force, $F_{t}$, is defined as,

$\vec{F}_{t}=-k_{B}TS_{T}\nabla T$ (4)

where $k_{B}$ is the Boltzmann constant, $T$ is the fluid temperature, $\nabla T$ is the temperature gradient, $S_{T}$ is the Soret coefficient. For PS particle, $S_{T}$ is positive of 18 K^-1^, which means most particles will migrate toward the colder region in a non-isothermal flow.

The drag force $F_{t}$is arising from thermal convection flow, which is defined by the Stokes drag law,

$\vec{F}_{d}=\frac{m_{p}}{\tau_{p}}(\vec{u}-\vec{v})$, (5)

where $u$ and $v$ are the fluid and particle velocities, respectively, $m_{p}$ is the microparticle mass, $\tau_{\text{p}}$is the characteristic time scale for a particle to accelerate due to the Stokes drag,

$\tau_{p}=\frac{2\rho_{p}r_{p}^{2}}{9\mu}$, (6)

in which $\rho_{p}$ is the density of particle, $\mu$ is the fluid dynamic viscosity, $r_{p}$ is the radius of particle. In our experiments, the density of particle ($r_{p}$=10 μm) is 1050 kg/m^3^, fluid dynamic viscosity is 0.001 Pa·s, and the characteristic time scale is about 26 μs. Because the particles are very small and its velocity relative to the fluid is small, the Stokes drag law is applicable.

Therefore, the trapping force$\vec{F}_{trap}$ of particles in aqueous solution is mainly composed of the horizontal thermophoretic and drag forces, neglecting gravity force,

$\vec{F}_{trap}=\vec{F}_{d}+\vec{F}_{t}$. (8)

**Minimum power selection**

We find that the change of power will have an effect on the trapping of particles in the thermo-optical tweezers. In Figures S2A-D, the experiment demonstrates how the position of the trapping potential well changes with optical power levels ranging from 0 mW to 7 mW. Figure S2E depicts the relationship between optical power and displacement. Particularly within the optical power range of 0 mW to 3 mW, significant fluctuations and changes in the trapping potential well's position are observed. As optical power increases further, the potential well's position gradually stabilizes after entering a stage of slow growth. This stable positioning enhances particle trapping stability and accuracy. To achieve optimal non-contact capture, it's essential to select the furthest distance possible while maintaining an appropriate optical power level. Within a certain range, increasing optical power can enhance the stability and depth of the trapping potential well, providing a more reliable trapping environment for particles. In our research, we typically utilize laser power exceeding 3 mW to ensure ample trapping power and stability for effective particle manipulation.

**
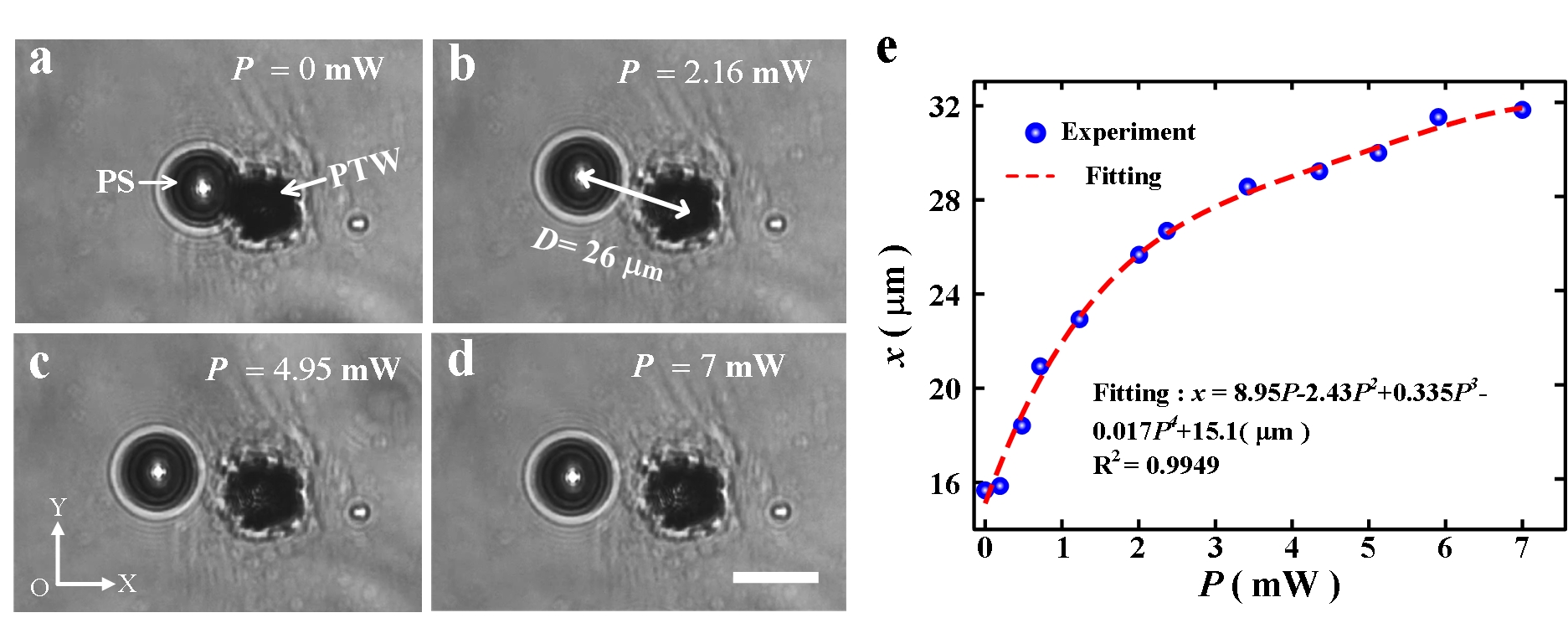
**

**Fig. S2. Experimental study on the manipulation of particles by thermo-optical tweezers at different optical powers.** (A-D) Particle manipulation experiment results under 0 ~ 7 mw optical power show that PS particles all move towards the trapping potential well, and the position of the trapping potential well changes with the change of optical power, where *D* represents the horizontal distance between the particle and PTW. (E) How the *x*-displacement of the particle varies with the optical power. Scale bars measure 20 μm.


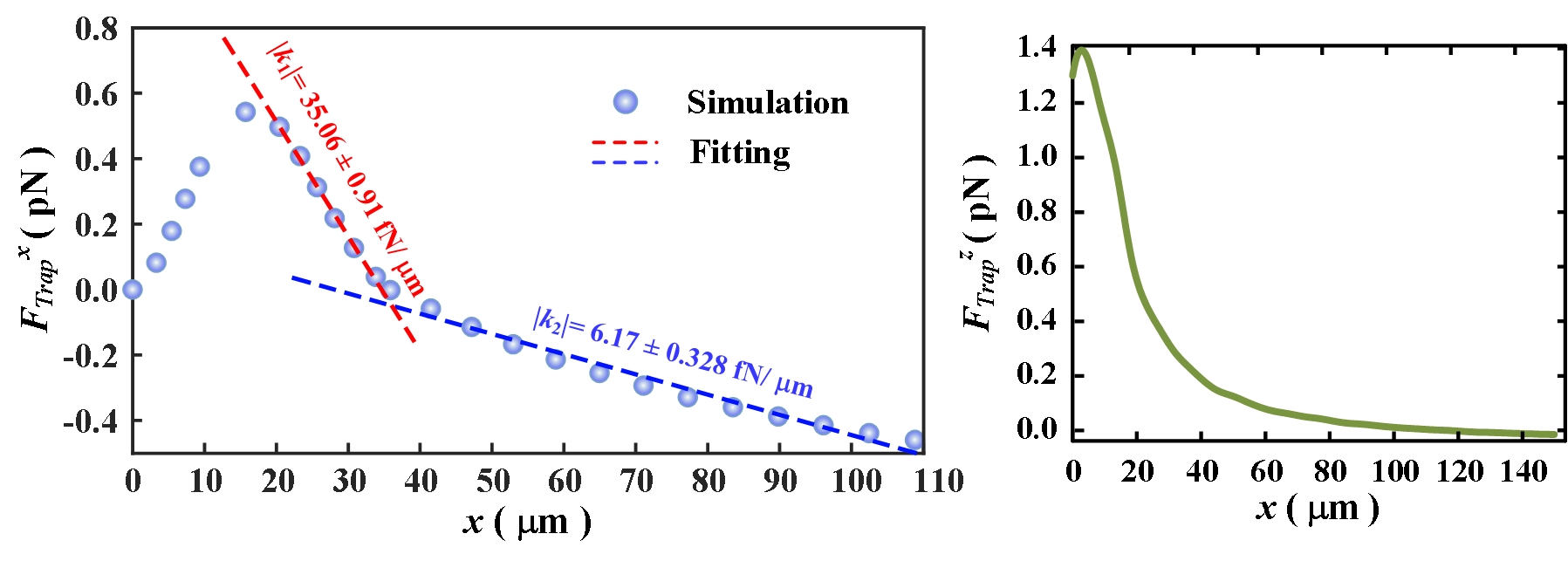


**Fig. S3. The trapping force-displacement relationship is used to measure the trap stiffness:** (A) The trap stiffness measurement curve of the fluid based traps, the positive and negative trap stiffness along the X-axis are *|k_1_|=* 35.06 ± 0.91 fN/µm and *|k_2_|=* 6.17 ± 0.328 fN/µm. (B) The combined force of thermophoresis and convection on z component of PS particle. The maximum force acting on the particle from the light source to the capture position is about 1.4 pN. After balancing the gravitational and buoyancy forces on the 20 μm PS microspheres, the calculated force is approximately -2.4 pN.

**
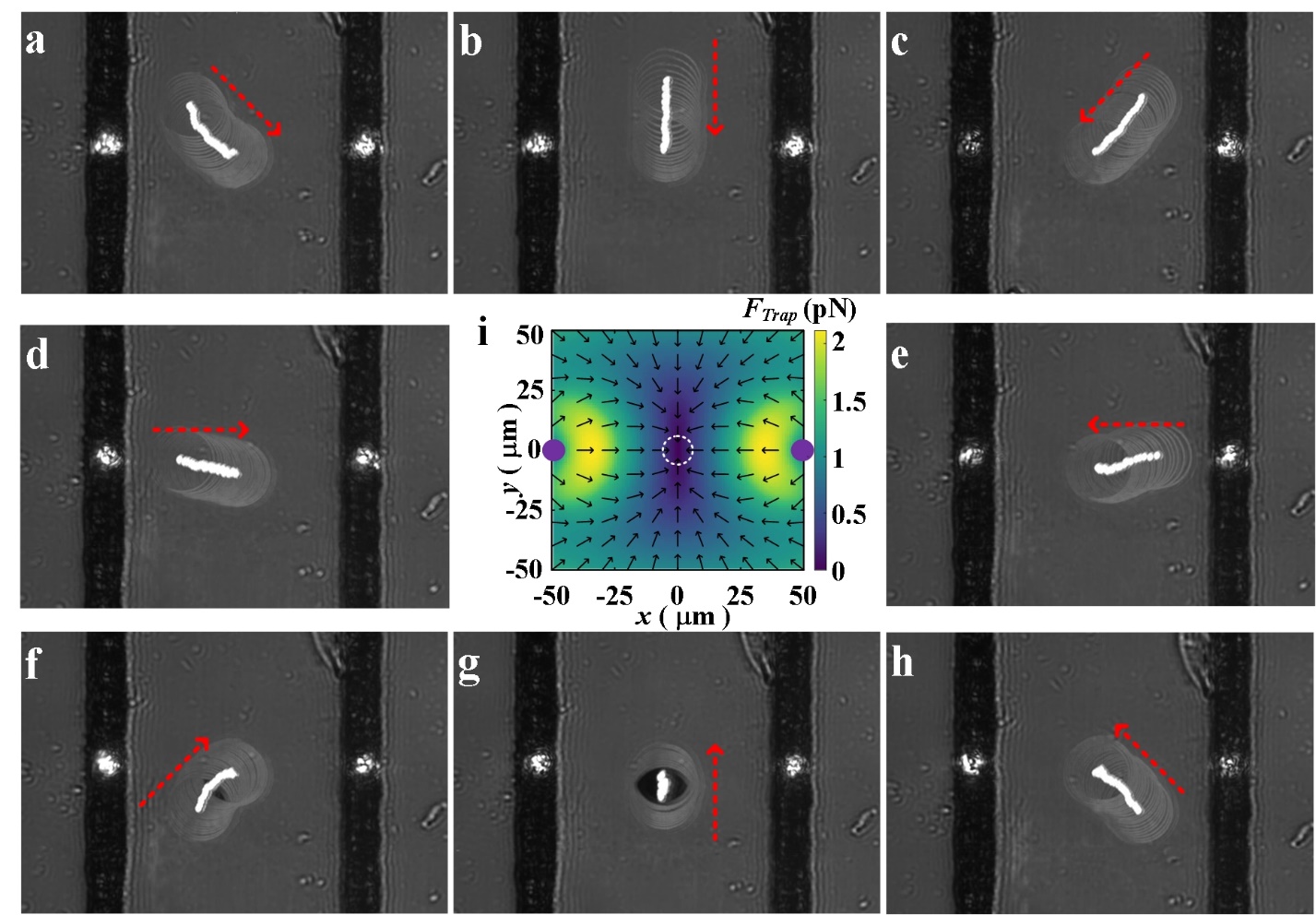
**

**Fig. S4. Dynamic manipulation of a single PS particle in different positions by DFLs.** (A)-(D), (E)-(H) The PS particles initially positioned in the upper left, upper, upper right, left, right, lower left, lower, and lower right directions all converge towards the central trapping potential well. (I) This section depicts the trapping force distributions on the *x*-*y* plane. The white dotted circle indicates the predicted center of the trapping potential well, while the blue dots at *y*=0 mark the positions of the double laser sources. The color bar illustrates the amplitude, and the arrows indicate the directions of the trapping force. The incident beam power of each laser is 16.0 mW, with a distance of 100 μm between the two tracks. Scale bars measure 25 μm.

**
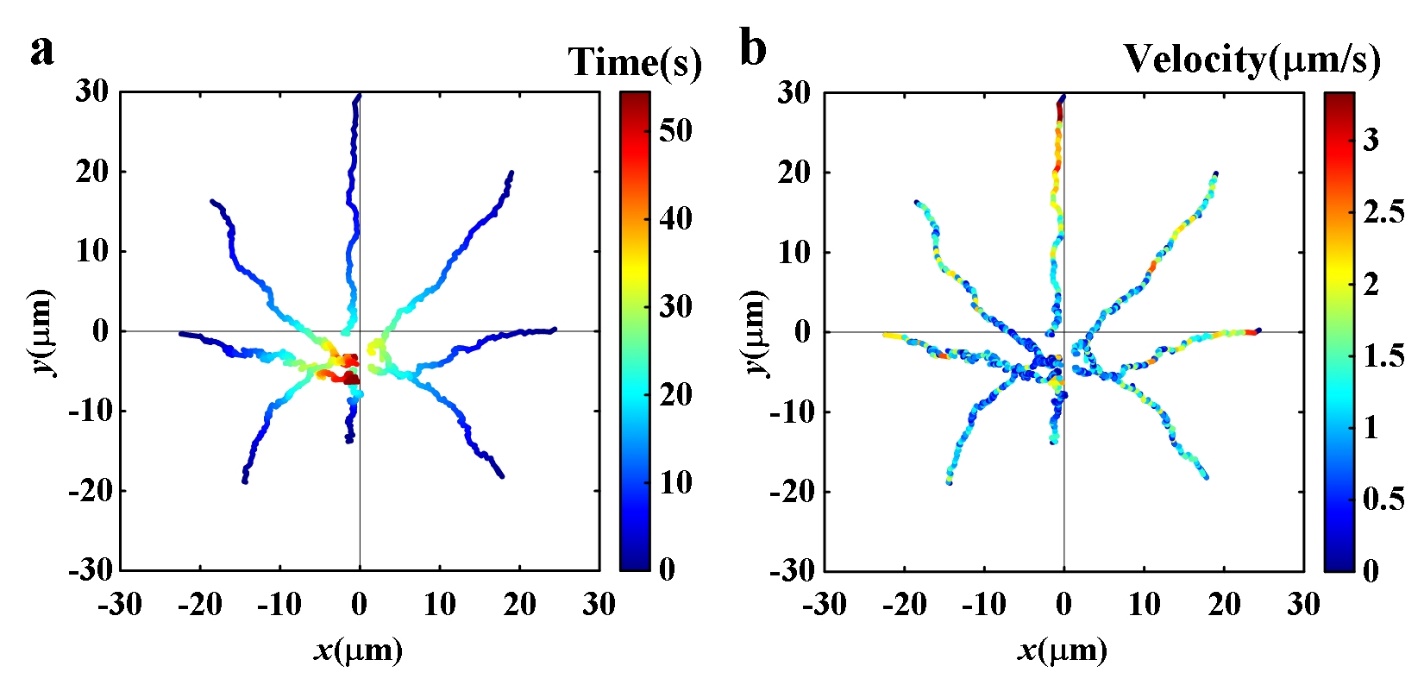
**

**Fig. S5**. **The trajectories vary with time for the PS particles moving from the upper left, upper, upper right, left, right, lower left, lower, and lower right directions to the trapping potential well (the center of the reticle).** (A) The particles ultimately stabilize near the center of the potential well. (B) The velocities of the PS particles, originating from various positions, vary over time. Initially, the velocities are relatively high at the start of their migration, but they slow down significantly upon nearing the center. This behavior aligns well with the distribution of the trapping force depicted in Fig. S2(I). The average velocity of the particles moving in the eight directions is approximately 0.87 μm/s. For a visual representation, experimental videos are provided in Movie S2.

**Negative index particle capture**

Regarding the control of particles with different refractive indices and irregular shapes, the refractive index of the fluorinated liquid microspheres in our study is 1.287, while the refractive index of the surrounding liquid (water) is 1.33. It can be observed that the refractive index of fluorinated liquid microspheres is significantly lower than that of water, indicating that PS microspheres are negative refractive particles. In optical tweezers particle manipulation, positively refractive particles are trapped at the laser focus, while negatively refractive particles experience repulsion under optical tweezers. The essence of the thermal optical tweezers proposed in our study is primarily to control the surrounding flow field variations by generating a thermal field with a laser, rather than directly acting on particles with light or heat fields. As shown in Figure S6A. It can be observed that within 0 to 50 seconds, fluorinated liquid particle were attracted and captured from a distance of 104 µm. Similarly, as depicted in Figure S7B, these particles were moved from the photothermal waveguide to the capture position within 0 to 1.5 seconds. Fully explain the thermal optical tweezers can capture particles without being affected by differences in particle refractive indices.


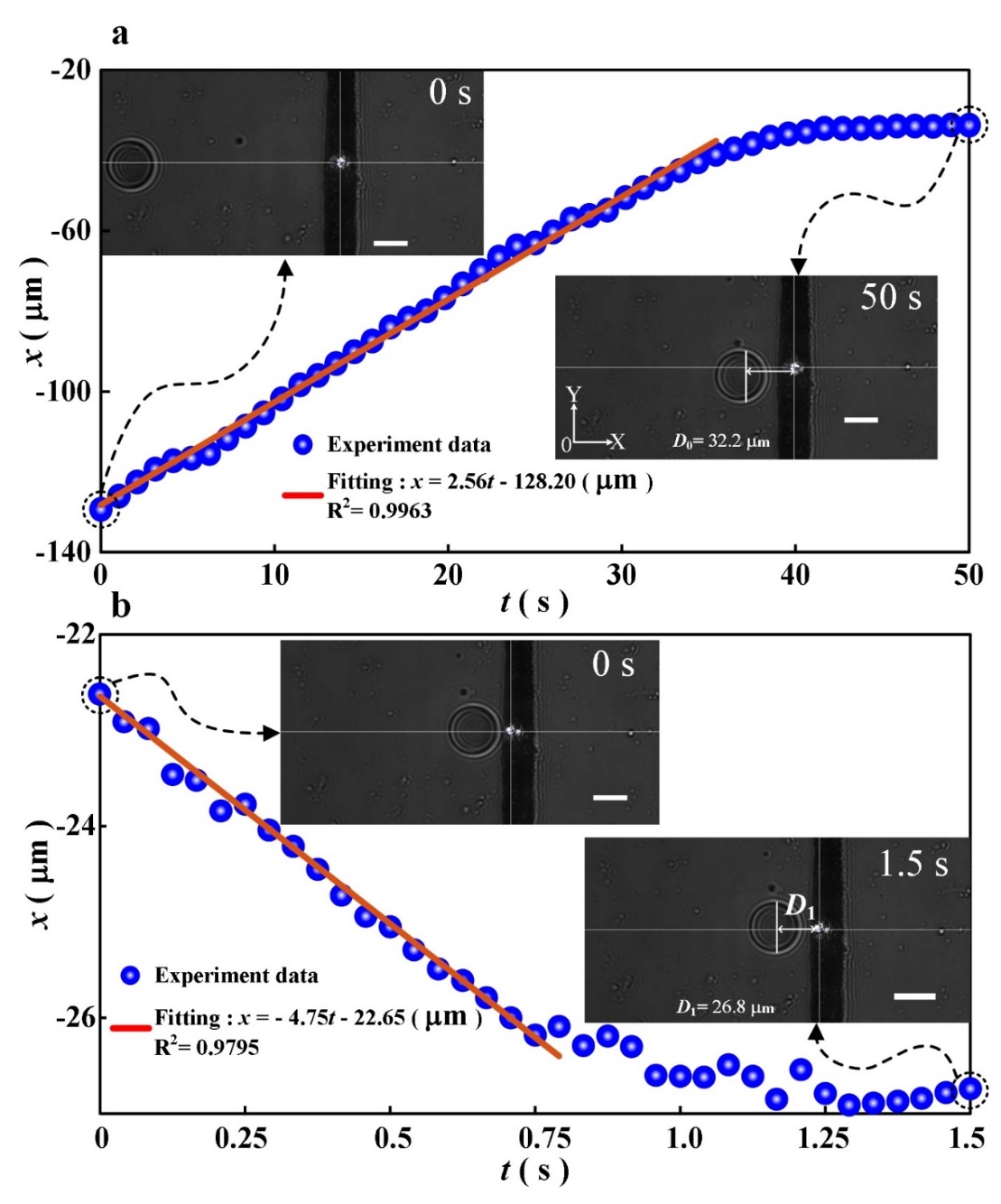


**Fig. S6**. **Dynamic manipulation of fluorinated liquid microparticles (diameter: 30μm) with negative refractive index by the TOT.** (A) The *x*-displacements of a distant fluorinated oil particle vary over time. This particle is attracted towards the trapping potential well. The red line represents a fit of the *x*-displacement of the fluorinated oil microparticle from 0 to 35 seconds. (B) The *x*-displacements of a nearby fluorinated oil particle also vary over time. In contrast, this particle is repelled away from the trapping potential well. The red line here fits the *x*-displacement of the fluorinated oil microparticle from 0 to 0.8 seconds. The incident beam power, measured at the rear aperture of the objective, is 16.1 mW. The scale bar measures 25 μm. The experimental video present this phenomenon is available in Movie

**Irregular disk particles capture**

Regarding the capture of non-spherical particles, we have performed experiments capturing irregular disk-shaped microparticles as shown in Figure S7A. It can be observed that within 0 to 40 seconds, irregular disk-shaped particle were attracted and captured from a distance of 80 µm. Similarly, as depicted in Figure S7B, these particles were moved from the photothermal waveguide to the capture position within 0 to 5 seconds. The variations in distance and time during their movement, illustrated in Figures S7C, D, show a predominantly linear motion before achieving stable capture. Different types of particles may exhibit varying effects during the capture process due to the influence of the captured material on the flow field. Spherical objects, owing to their high degree of symmetry, have a smaller impact on the flow field. In contrast, the irregular particles shown in Figure S7 feature an axial distribution of lengths and breadths. The capturing potential wells formed by the photothermal tweezers, as depicted in the inset of Figure 3a, demonstrate a greater lateral distribution along the y-axis at the capture location. Consequently, irregular particles exhibit deflection upon reaching the capture point, aligning their long axes along the y-axis. Subsequent experiments indicated a longitudinal distribution of particles, as shown in Figure S7B, The particles consistently displayed a longitudinal distribution, and the capture site also underwent changes due to their morphological transformation. Overall, the capture morphology is related to the initial state of the particles being captured. Irregular objects may undergo some rotational manipulations, but these may not be sustainable.


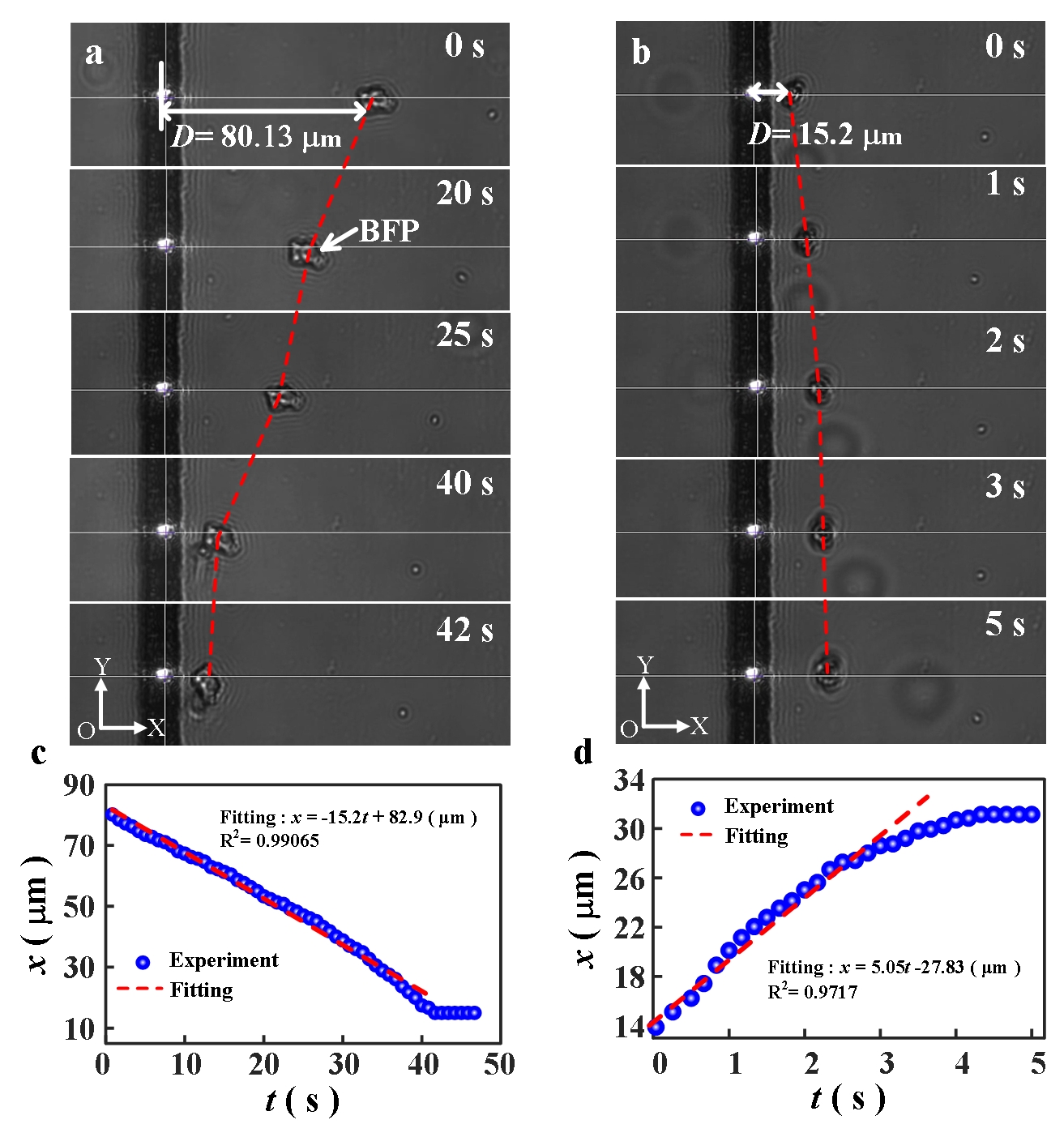


**Fig. S7. Dynamic manipulation of irregular disk particles represented by bamboo fiber particles (BFP), by the TOT.** (A) The distant irregular BFP material in the 0 s -40 s is attracted to the trapping potential well. D is the *x* displacement of the irregular disk particle to the single-focused laser. (B) Nearby irregular BFP material is repelled to the annulus trap potential well. (C) The *x* displacement of an irregular BFP substance changes with time in the attractive state, and (D) the *x* displacement of an irregular disk particle changes with time in the repulsive state. The trajectory where the BFP is captured to the well is represented by a red dashed line, and the incident beam power (measured at the aperture behind the objective) is 12.1 milliwatts. Scale :25 μm. The experimental video present this phenomenon is available in Movie S9.


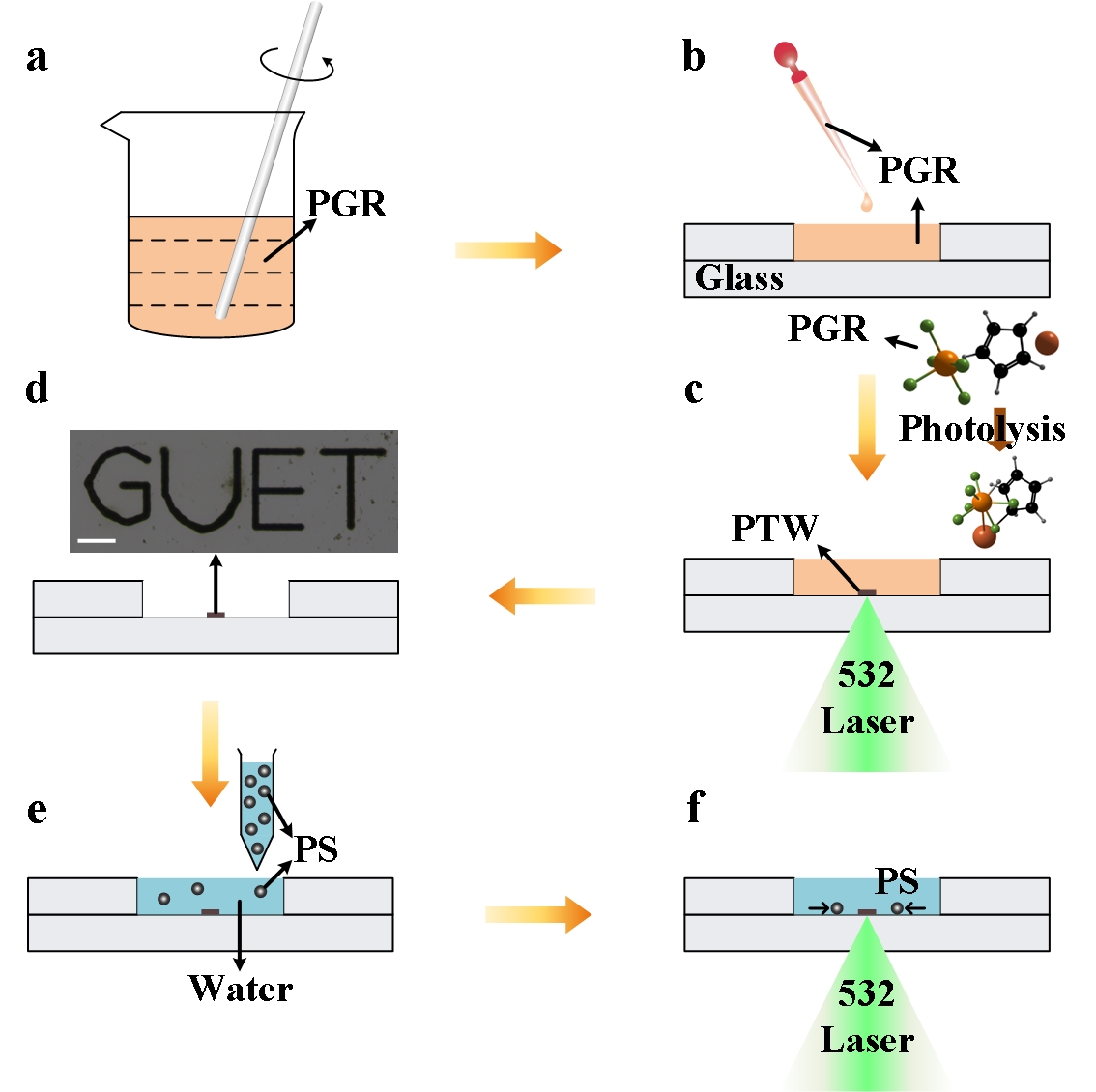


**Fig. S8. Synthesis of PTW and sample preparation.** (A) Preparation of PGR solution. (B) Dropping the PGR solution into the micro-channel. (C) Focusing the laser on the bottom of the micro-channel, which triggers the PGR to self-assemble into a crystalline structure, forming a solid PTW. (D) Engraving a 'GUET' patterned structure of PTW with a scale of 100 μm. (E) Introducing an aqueous solution of PS particles into the micro-channel. (F) Utilizing the TOT system to manipulate microparticles.
